# A Cognitive Study of Dietary Imbalance Behaviors in Chinese Multigenerational Households Based on the Information–Motivation–Behavioral Skills Model

**DOI:** 10.3390/nu18162687

**Published:** 2026-08-18

**Authors:** Yue Ding, Chun Yang, Rong Deng

**Affiliations:** School of Design, Jiangnan University, Wuxi 214122, China; 6240310007@stu.jiangnan.edu.cn (Y.D.); 8202201014@jiangnan.edu.cn (C.Y.)

**Keywords:** extended family, dietary imbalance, information–motivation–behavioral skills model, false consensus effect, cognitive dissonance

## Abstract

**Background/Objectives**: Family dietary imbalance is an important source of the double burden of malnutrition, and dietary arrangements in multigenerational households are often deeply embedded in intergenerational caregiving relationships. However, the cognitive formation mechanism through which dietary caregiving behaviors driven by care and responsibility may instead lead to dietary imbalance still lacks systematic explanation. Therefore, this study aimed to examine the cognitive formation mechanism of dietary imbalance behaviors in Chinese multigenerational households. **Methods**: This study takes primary dietary caregivers in Chinese three-generation households as the research subjects. It integrates the false consensus effect, cognitive dissonance theory, and the information–motivation–behavioral skills (IMB) model. Based on 467 valid samples, the study conducts empirical analysis using partial least squares structural equation modeling (PLS-SEM) and importance-performance map analysis (IPMA). **Results**: The results show that caregivers’ subjective judgments about family members’ dietary needs based on their own experience trigger cognitive dissonance and further promote their re-examination of dietary risk information, caregiving responsibilities, and family normative expectations. Among these factors, caregiving responsibilities and subjective norms are key factors in improving dietary regulation self-efficacy, whereas the effect of risk information perception on self-efficacy is not significant. The IPMA results further show that self-efficacy is the most important factor driving the formation of behavioral intention to regulate healthy family diets. **Conclusions:** This study reveals the psychological mechanism through which dietary imbalance behavior in multigenerational households moves from subjective need judgment to the formation of future regulation intention. It extends the application of the IMB model in family caregiving contexts and provides a theoretical basis and practical implications for the formulation of family health promotion and dietary intervention strategies.

## 1. Introduction

Dietary imbalance is widespread in the global population. It can cause malnutrition and other problems and pose serious threats to health. Member states of the United Nations have made the elimination of all forms of malnutrition an important part of the global agenda [1]. Imbalanced nutritional structure not only directly affects individual growth and development, chronic disease risk, and quality of life, but also substantially increases the disease burden and resource pressure on global public health systems. It is regarded as one of the major challenges currently facing global public health [2].

In China, imbalanced family dietary structure is particularly prominent, and nutritional risk differs markedly across age groups. On the one hand, China faces a serious problem of population aging. Older adults account for 18.7% of the population, and nearly half of them are malnourished [3]. On the other hand, the prevalence rates of overweight and obesity among children under 6 years old and adolescents aged 6–17 in China have reached 10.4% and 19.0%, respectively.

Relevant research has shown that adolescents and young adults (AYAs) worldwide exhibit unhealthy dietary behaviors, and these behaviors are influenced by multilevel contextual factors, including interpersonal relationships [4]. Family resilience and social support are also strongly positively correlated with individual quality of life [5]. The widespread existence of multigenerational households means that dietary behaviors and nutritional outcomes among members of different generations are not independent of one another [6,7]. Instead, they influence each other through co-residence and daily interaction. This introduces a more diverse research perspective for age-related nutritional differentiation and makes it necessary to conduct in-depth research on this typical family structure.

Multigenerational households are defined as households in which grandparents (the first generation), parents (the second generation), and grandchildren (the third generation) live together [8]. In China in particular, three-generation household life is common [5].In multigenerational households, eating unhealthily out of caregiving constitutes a common but often overlooked dietary phenomenon. It is manifested when caregivers, driven by caregiving motives, express responsibility and care by intervening in eating practices. This caregiving motive is oriented towards altruistic behavior and is closely related to long-formed notions of thrift and role identity. Although this phenomenon is often summarized through a specific generational image, the related dietary behaviors are not limited to one age group; they may appear among different family members who undertake caregiving responsibilities [6,7,8]. Relevant studies have indicated that adult roles in multigenerational families may all undertake caregiving for older adults [8,9]. Existing research has revealed the influence of grandparental and parental caregiving behaviors on children’s dietary and nutritional outcomes [6], the mechanism by which care-driven behavior leads to household food waste [8], and the family-level double burden of malnutrition. However, why caregiving behaviors motivated by care and responsibility may instead lead to dietary imbalance in multigenerational households has not been fully explained. Therefore, this study focuses on the primary dietary caregivers in families, referring to family members who primarily undertake family meal arrangement, food provision, and dietary caregiving responsibilities in multigenerational households, hereafter abbreviated as caregivers.

This study has multiple theoretical and practical implications. First, unlike existing studies that mainly begin with individual nutritional choices, this study uses three-generation households as the unit of analysis and systematically reveals the formation logic of dietary imbalance behavior in caregiving contexts. It thereby expands understanding in family dietary behavior research of the paradox between caregiving motives and health outcomes. Second, by introducing social psychological theories such as cognitive dissonance and the false consensus effect and integrating them into the information–motivation–behavioral skills (IMB) theoretical framework, this study explains, at the mechanism level, how caregivers make dietary decisions that deviate from health goals under well-intentioned caregiving motives and gradually form future regulation intentions through subsequent feedback. This further enriches the application of health behavior theory in family caregiving contexts.

This study takes caregivers in multigenerational households as the object of analysis and constructs and tests the formation mechanism of the dietary imbalance phenomenon of eating unhealthily out of caregiving. The study integrates the IMB model, the false consensus effect, and cognitive dissonance theory, and conducts empirical analysis using partial least squares structural equation modeling. The article is organized as follows. Section 2 reviews research progress on the IMB model, the false consensus effect, and cognitive dissonance, and develops the research model and hypotheses. Section 3 introduces materials and methods. Section 4 evaluates the measurement model and structural model and presents the empirical results through importance-performance map analysis (IPMA). Section 5 discusses the findings. Section 6 discusses theoretical and managerial contributions, limitations, and future research directions.

## 2. Literature Review and Research Hypotheses

### 2.1. Information–Motivation–Behavioral Skills Model

The theoretical foundation of the IMB was formed in Fisher and Fisher’s series of studies on AIDS risk behavior. In subsequent research, it was explicitly named and empirically tested as the information–motivation–behavioral skills model [10], and gradually developed and improved as a behavioral explanatory framework.

The model indicates that the formation of individual health behavior is usually associated with three core elements: information, motivation, and behavioral skills. Information refers to an individual’s understanding of health risks and behavioral consequences. Motivation includes individual attitudes and normative influences from important others. The IMB model uniquely combines personal motivation and social motivation with behavioral skills, dividing motivation into personal and social aspects. Personal motivation reflects an individual’s attitude toward self-management, whereas social motivation includes external support from others [11]. Behavioral skills, including self-efficacy, involve the practical abilities needed to implement behavior, such as verbal and nonverbal abilities for communication, negotiation, and refusal. They also include an individual’s confidence judgment about his or her own ability and are crucial for effectively completing self-management tasks [10,11]. Relevant studies further indicate that information and motivation often affect behavior through behavioral skills rather than directly influencing behavioral outcomes [10].

However, the IMB model mainly focuses on the formation of behavioral intention. It provides relatively limited explanation of how individuals form judgments about others’ needs before a behavior occurs and how they make cognitive adjustments after the behavior has been implemented. Therefore, it is necessary to introduce upstream cognitive mechanisms to supplement this framework.

### 2.2. Meanings of the IMB Model Variables in This Study

In the context of family dietary caregiving, the three dimensions emphasized by the IMB model, namely information, motivation, and behavioral skills, need to be further specified in relation to the specific behavioral context.

At the information level, individual behavior is usually influenced by judgments about health status. Su et al. [12] found that individuals’ perceived severity of health risks is positively correlated with self-efficacy, and that self-efficacy affects health risk prevention behaviors. In multigenerational households, caregivers form judgments about possible dietary imbalance among family members and its potential health consequences through daily interaction and accumulated experience. Zhang et al. [13] further showed that perception of disease risk information and self-efficacy are important factors promoting health behavior. In addition, the effect of such risk perception on health behavior is mediated by self-efficacy.

At the motivation level, the IMB model understands behavioral motivation as jointly composed of personal motivation and social motivation. In family contexts, individual decision-making is influenced not only by personal attitudes but also by role responsibility and social norms. On the one hand, caregivers form responsibility cognition based on family roles. Zhang et al. [14] noted that caregivers’ filial piety beliefs are an important component of the caregiving process, including burden, coping, and seeking information and support, in many cultures. They are also an important factor influencing caregiver self-efficacy, and a significant association between caregiving burden and caregiver self-efficacy has been confirmed. Teng et al. [15], in a study related to caregivers’ general self-efficacy, found that cultivating caregivers’ sense of identity and responsibility can enhance their positive emotions and confidence. On the other hand, caregivers also perceive normative expectations from family members and sociocultural contexts. In research on motivation shaping healthy eating intentions, Ma et al. [16] indicated that individuals may strengthen their intention to participate in a behavior because they hold a positive attitude toward it or feel social pressure. The study confirmed that attitude and subjective norms are significantly related to intentions, and that motivation strongly influences behavioral intention for healthy eating.

At the behavioral implementation level, the IMB model emphasizes the abilities needed for individuals to implement behavior and their confidence judgments about their own abilities. As an important manifestation of this ability judgment, self-efficacy plays a key role in the formation of behavioral intention. Previous studies have shown that attitudes, subjective norms, and perceived behavioral control directly influence proactive health behavioral intention and also exert indirect effects through the mediating role of self-efficacy [17]. Han et al. found that individuals’ intrinsic altruistic motivation provides the fundamental driving force for altruistic behavior, whereas its translation into actual action is guided by perceived general self-efficacy [18]. Zhang et al. further demonstrated that self-efficacy not only mediates the relationship between caregiver burden and caregiver gain among Chinese caregivers but may also facilitate the transformation of caregiver burden into caregiver gain [14]. Wang et al. also confirmed that strengthening risk perception and self-efficacy can promote proactive health behaviors and that incorporating self-efficacy into behavioral interventions facilitates the adoption of healthier behaviors [19].

### 2.3. False Consensus Effect and Cognitive Dissonance

The false consensus effect (FCE) is an important theory in social psychology used to explain systematic bias in individuals’ social judgment processes. The theory refers to a cognitive bias whereby individuals tend to mistakenly assume that others share the same attitudes, views, and beliefs as themselves. They overestimate consistency between others and themselves and thereby project their own cognition onto others [20].

In family contexts, when judging family members’ needs, caregivers tend to overestimate the universality of their own cognition, thereby producing biased need judgments [8]. Further, this cognitive bias may be relatively pronounced in contexts involving judgments or decisions made on behalf of others. When individuals make judgments based on limited information, they are more likely to rely on their own experience to infer.

Existing studies show that when behaviors implemented based on such judgments fail to achieve expected results, individuals are more likely to become aware of inconsistency between cognition and reality and to experience cognitive conflict [8]. This process constitutes an important source of cognitive dissonance.

Cognitive dissonance (CD), proposed by Festinger, explains the psychological discomfort individuals experience when their cognitions or behaviors are inconsistent [8]. Relevant research indicates that cognitive conflict is not only manifested as psychological discomfort, but may also prompt individuals to reprocess information and adjust behavioral judgments [8]. In the context of family dietary caregiving, when the actual outcomes of caregiving behavior are inconsistent with original judgments, individuals may reassess health risks, responsibility cognition, and normative expectations, thereby influencing subsequent behavior. Liu et al. [8] also noted that when caregivers already know that the behavior they insist on contradicts long-formed values but still choose to do it, cognitive dissonance negatively affects the social expectations caregivers perceive from family and peers, as well as the personal attitudes and emotional responses related to caregiver motivation. The cognitive dissonance context in the present study is the opposite. It is a state of psychological disharmony caused by inconsistency between caregivers’ caregiving intentions and actual results, in which caregivers are uncertain whether their behavior is correct.

Therefore, cognitive dissonance in this study is not used to explain why caregivers initially form subjective judgments. Instead, it explains how caregivers enter a process of reflection and reassessment after such judgments are shaken by real-world feedback. When caregivers form strong expectations based on subjective need judgments, once actual feedback is inconsistent with those expectations, their original cognition is more likely to be challenged, thereby producing cognitive dissonance.

### 2.4. Development of Research Hypotheses

The formation of family dietary regulation behavior is not directly determined by a single factor. Rather, it is more likely to appear as a continuous process from need judgment to cognitive change and then to behavioral intention formation. In multigenerational households, caregivers often first form judgments about family members’ dietary needs based on their own understanding and arrange diets accordingly. When real-world feedback enters the caregiving process, caregivers may re-examine their original arrangements and further change their understanding of dietary risk information, caregiving responsibility, and family expectations.

**Figure 1:**
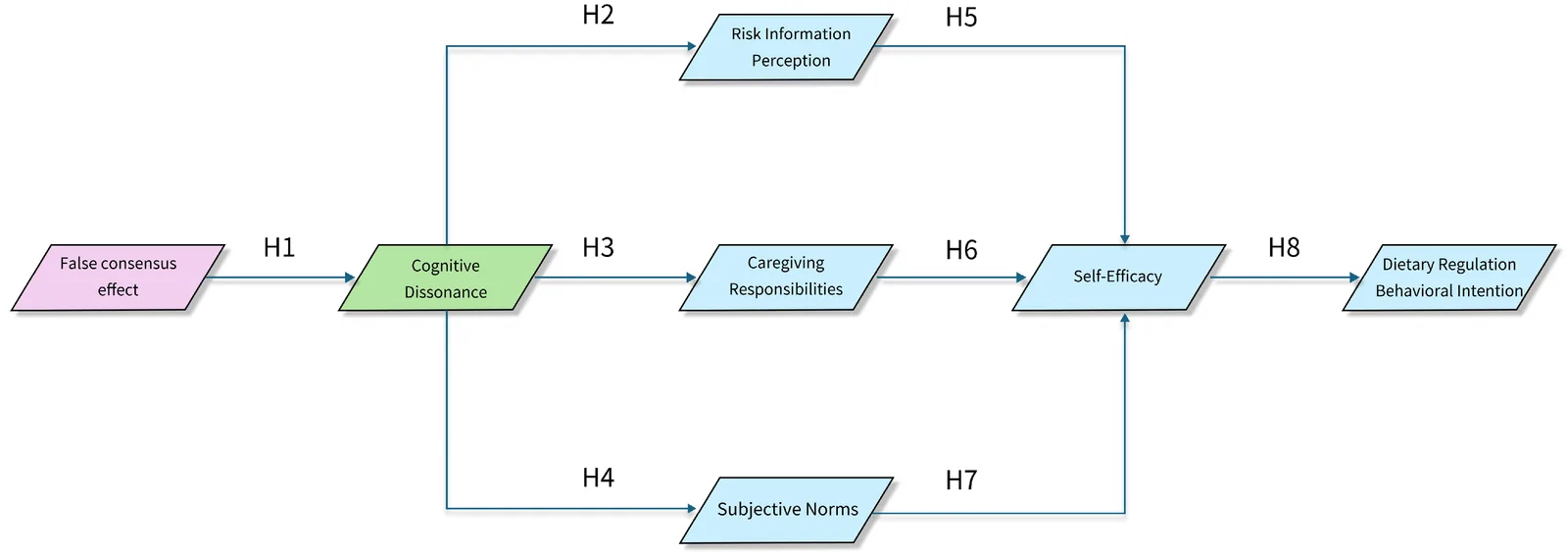
Research Model.

On this basis, caregivers’ cognition of health risks, perceptions of family responsibility and normative expectations, and confidence judgments about their own ability to implement dietary communication and adjustment may jointly enter the behavioral intention formation process and further influence their future behavioral tendency to regulate family diets. Therefore, based on the overall logic of need judgment, cognitive change, and behavioral intention formation, this study integrates the relationships among the false consensus effect, cognitive dissonance, and variables related to the IMB model, and proposes the following research hypotheses (Figure 1):

**H1.**  *The false consensus effect positively affects cognitive dissonance.*

**H2.**  *Cognitive dissonance positively affects risk information perception.*

**H3.**  *Cognitive dissonance positively affects caregiving responsibilities.*

**H4.**  *Cognitive dissonance positively affects subjective norms.*

**H5.**  *Risk information perception positively affects self-efficacy.*

**H6.**  *Caregiving responsibilities positively affect self-efficacy.*

**H7.**  *Subjective norms positively affect self-efficacy.*

**H8.**  *Self-efficacy positively affects dietary regulation behavioral intention.*

## 3. Materials and Methods

### 3.1. Scale Design

Based on existing mature scales, this study adaptively revised the items in combination with the context of dietary caregiving in multigenerational households. After the questionnaire was formed, some expressions were further adjusted based on expert opinions and pre-survey results.

The FCE and CD scales refer to the study by Liu, Zhu, Wang, and Wu [8]. FCE mainly measures caregivers’ tendency to infer family members’ dietary needs based on their own experience and subjective understanding. The relevant items involve dietary experience judgment, emotional investment, and subjective grasp of family dietary arrangements. CD mainly includes caregivers’ uncertainty and tendency to rethink current dietary arrangements when dietary caregiving outcomes do not meet original expectations. The risk information perception (RIP) scale refers to relevant studies by Vu, Nguyen, Vu, Tran, and Vu [21] and de Andrés-Sánchez, Puelles-Gallo, Souto-Romero, and Arias-Oliva [22]. It mainly covers the cognitive judgments and risk concerns formed by caregivers after receiving feedback information related to family diets regarding possible risks in the family’s current dietary status and its potential health consequences. The measurement of caregiving responsibilities (CR) was adapted from the studies by Landi, Boccolini, Giovagnoli, Pakenham, Grandi, and Tossani [23] and Ha, Różycka-Tran, Jurek, Thu, and Hao [24], involving family responsibility awareness, proactive care, and participation in daily dietary caregiving. Subjective norms (SN) refer to the relevant scale of Liu, Zhu, Wang, and Wu [8] and mainly measure the normative influences perceived by caregivers during family dietary caregiving, including family expectations, social beliefs, and attitudes of important others. The self-efficacy (SE) scale refers to the study by Romppel, Herrmann-Lingen, Wachter, Edelmann, Düngen, Pieske, and Grande [25] and was adjusted in combination with the family dietary regulation context. It mainly includes confidence in abilities related to dietary communication, problem response, and family dietary coordination. The measurement of dietary regulation behavioral intention (DRBI) refers to the studies by Ma, Lee, and Hwang [16] and Li and Shan [26], covering behavioral willingness to actively conduct family dietary communication, adjust dietary arrangements, and continuously carry out dietary regulation in the future. All items were measured using a seven-point Likert scale, where 1 indicates strongly disagree and 7 indicates strongly agree. The measurement items and sources for each variable are shown in Table 1.

### 3.2. Settings and Participants

This study collected data through an online questionnaire. The questionnaire consisted of three parts: (1) research description, (2) respondents’ basic information, and (3) main survey items. The research description mainly introduced the research purpose, completion requirements, and anonymity principle to respondents. The basic information section covered demographic characteristics such as age, gender, education, and household income level. The main survey items focused on the constructs in the theoretical model: false consensus effect, cognitive dissonance, risk information perception, caregiving responsibilities, subjective norms, self-efficacy, and behavioral intention to regulate healthy family diets. It should be noted that this study employed a cross-sectional survey design. Therefore, the analysis focused on theoretically grounded relationships among the constructs rather than changes in these constructs over time, and causal conclusions cannot be drawn from the results [27].

Data were collected from March to April 2026 through the Micro Survey platform, and a total of 500 questionnaires were obtained. Given the focus on dietary caregiving behavior in multigenerational households, predefined eligibility criteria were applied before participation. Eligible respondents were required to be aged 20 years or above, married, and parents, and to belong to families in which three generations lived together or maintained frequent and long-term interactions. They were also required to be primarily responsible for family meal arrangement, food provision, or dietary caregiving. The platform screened potential respondents according to these criteria and invited eligible individuals to participate. Upon entering the questionnaire, respondents were presented with the study description, participation requirements, and information regarding anonymity and data use. They were informed that the data would be used solely for academic research, that responses would be analyzed anonymously, and that participation was voluntary, with the right to withdraw at any time. To enhance sample diversity, the questionnaire was distributed across different regions of China, with efforts made to include respondents from a range of educational backgrounds and household income levels. These procedures were intended to ensure that the characteristics of the participants were well aligned with the research context of dietary caregiving in multigenerational households.

After the questionnaires were collected, the sample data were further screened. Questionnaires with obviously abnormal response times, continuous selection of the same option, or obvious logical contradictions between earlier and later answers were excluded to improve data quality and result reliability. Finally, 467 valid samples were obtained, with a valid questionnaire rate of 93.4%. The specific demographic characteristics of the sample are shown in Table 2.

Male respondents accounted for 41.33% of the sample, while female respondents accounted for 58.67%. This gender distribution may reflect the diversity of dietary caregiving roles in multigenerational households. In such households, caregiving responsibilities are often shared among family members from different generations, embedding family dietary practices within everyday interactions and the division of caregiving roles [8]. Previous studies have shown that although women continue to undertake a larger share of household meal-related work, men’s involvement in family dietary caregiving has gradually increased, with many men assuming responsibilities equal to or greater than those of their partners in meal planning, grocery shopping, and cooking [28,29]. In Chinese families, fathers also participate in daily caregiving, household tasks, and family decision-making, and some assume primary caregiving responsibilities. Ma et al. further reported that in Chinese three-generation households, both grandparents and parents undertake dietary caregiving responsibilities, including food purchasing and meal preparation [30].

In addition, the gender composition of the sample was also influenced to some extent by the participant selection criteria, which were based on respondents’ actual dietary caregiving responsibilities. Because this study focused on individuals who actually undertook family dietary caregiving responsibilities rather than restricting eligibility to a particular gender or family role, eligible participants included members of different genders and generations.

### 3.3. Research Method

This study uses partial least squares structural equation modeling (PLS-SEM) as the main analytical method to test the relationships among the latent variables. This method is suitable for research models that include multiple latent variables and complex path relationships, and it can effectively handle predictive analysis problems in behavioral research. The model includes two parts: the measurement model and the structural model. First, the measurement model was tested for reliability and validity to evaluate the stability and consistency of each latent variable measurement. Subsequently, the structural model was evaluated. R^2^ and Q^2^ indicators were used to test the explanatory power and predictive power of the model, and the research hypotheses were verified through path analysis. In addition, to further compare the effects of different variables on the outcome variable, this study used IPMA to analyze the relative importance of the constructs.

## 4. Results

### 4.1. Measurement Model Evaluation

This study used SmartPLS 4 for statistical analysis. The analysis included items, factor loadings, Cronbach’s alpha (α), composite reliability (CR), and average variance extracted (AVE) for each construct [31].

As shown in Table 3, the standardized outer loadings of all measurement items ranged from 0.709 to 0.878, all above 0.7 [32], indicating that the items can effectively reflect their corresponding latent variables. The Cronbach’s α values of the latent constructs ranged from 0.700 to 0.875, demonstrating satisfactory internal consistency overall. Specifically, the Cronbach’s α value for the false consensus effect (FCE) construct was 0.700, meeting the commonly accepted threshold of 0.70 and representing the lower bound of acceptable reliability. CR ranged from 0.833 to 0.915, all above 0.70 [33], meeting the reliability requirement. Further, the AVE values of the latent variables ranged from 0.574 to 0.728, all exceeding the criterion of 0.50, indicating good convergent validity.

Next, discriminant validity was evaluated using two methods: the heterotrait–monotrait ratio (HTMT) and the Fornell–Larcker criterion. Table 4 shows that the HTMT values among all latent variables were below 0.90, indicating discriminant validity [34]. Table 5 reports the Fornell–Larcker test results. The square roots of AVE exceeded the corresponding inter-construct correlations in their rows and columns, further supporting discriminant validity [31].

### 4.2. Structural Model Assessment

Table 6 presents R^2^ and Q^2^. R^2^ reflects explained variance, whereas Q^2^ is used to assess the model’s predictive relevance. First, the R^2^ values of the endogenous variables ranged from 0.179 to 0.499. Specifically, the R^2^ values for CD, RIP, CR, SN, SE, and DRBI were 0.256, 0.179, 0.258, 0.212, 0.499, and 0.464, respectively, indicating that the model has a certain explanatory power for the endogenous variables. Second, the Q^2^ values for all variables were greater than 0, indicating that the model has good predictive relevance. The above results indicate that the model has good overall fit.

Bootstrapping was then used to test the hypotheses, as shown in Table 7. FCE had a significant positive effect on CD (β = 0.506, *p* = 0.000), supporting H1. CD had significant positive effects on RIP, CR, and SN (CD → RIP: β = 0.423, *p* = 0.000; CD → CR: β = 0.508, *p* = 0.000; CD → SN: β = 0.461, *p* = 0.000), supporting H2, H3, and H4.

**Figure 2:**
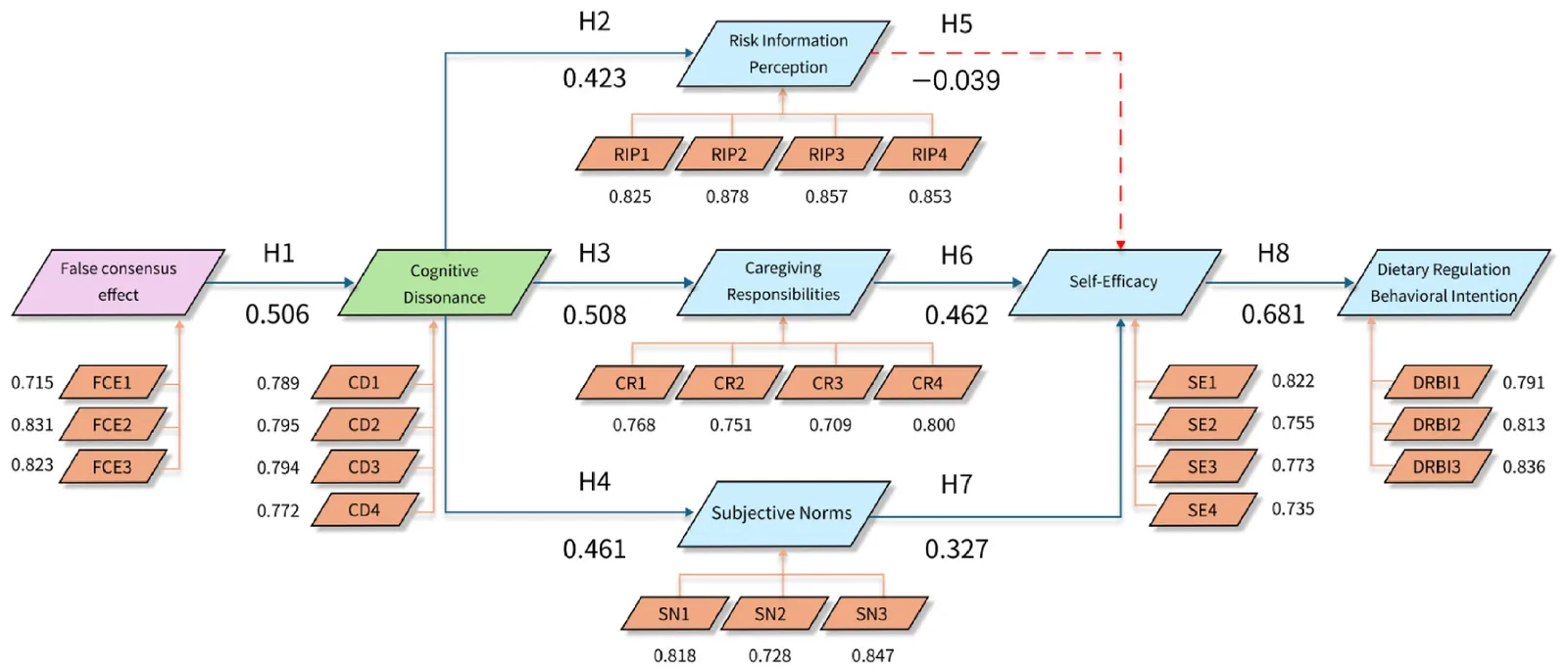
Analysis Results of the Hypothesized Model.

In the formation path of SE, CR and SN both had significant positive effects on SE (CR → SE: β = 0.462, *p* = 0.000; SN → SE: β = 0.327, *p* = 0.000), supporting H6 and H7. However, the effect of RIP on SE did not reach a significant level (RIP → SE: β = −0.039, *p* = 0.297), so H5 was not supported. In addition, SE had a significant positive effect on DRBI (SE → DRBI: β = 0.681, *p* = 0.000), supporting H8 (Figure 2).

In Table 7, the VIF values for all paths were below the conventional multicollinearity threshold (usually <10), indicating that the model does not have a serious collinearity problem [31].

### 4.3. Importance-Performance Map Analysis

IPMA is a method for simultaneously examining construct importance and performance levels based on PLS-SEM results. The horizontal axis represents the importance value of antecedent constructs, namely their total effect on the target construct, and the vertical axis represents their performance value, standardized on a 0–100 scale [31,35]. IPMA compares the total effects that represent the importance of antecedent constructs in forming a target construct with the average latent variable scores that represent their performance. The aim is to identify antecedent variables that have relatively high importance for the target construct, that is, constructs with stronger total effects, while their current performance levels are relatively low, that is, their average latent variable scores are low.

**Figure 3:**
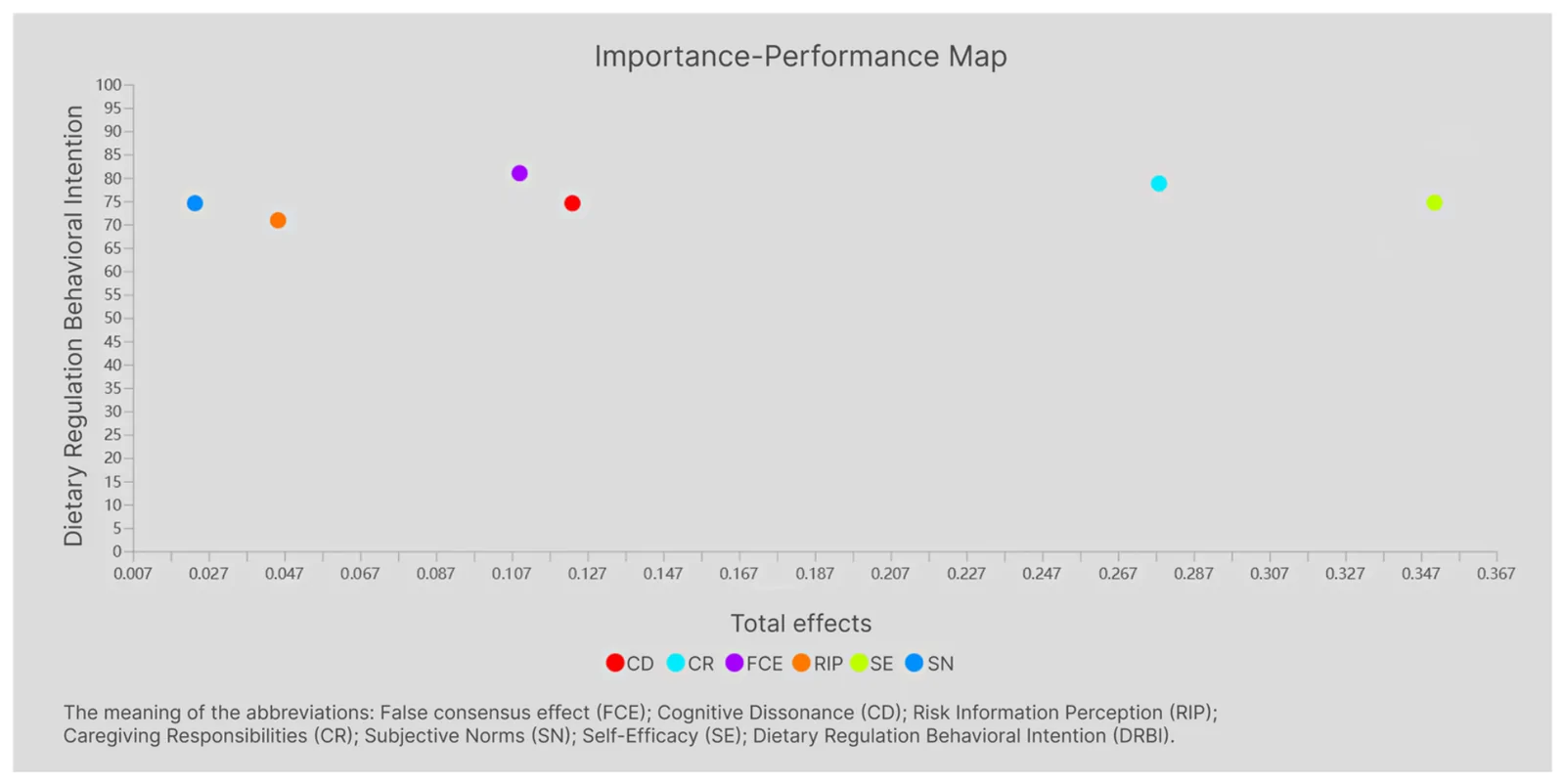
Importance-Performance Map.

Figure 3 shows that, in terms of importance, SE has the largest effect on DRBI, followed by CR, CD, and FCE, whereas RIP and SN have relatively low importance. In terms of performance, FCE has the highest score; CR, SE, CD, and SN are all at medium levels; and RIP is the lowest. These results indicate that self-efficacy is the key driver promoting the formation of dietary regulation behavioral intention, whereas risk information perception does not play a significant role through the self-efficacy path.

## 5. Discussion

Based on the IMB model and the perspectives of the false consensus effect and cognitive dissonance, this study constructed a model of dietary regulation behavioral intention in multigenerational households and used PLS-SEM and IPMA to analyze the formation process of family dietary regulation behavioral intention. The findings reveal the association mechanism among caregivers’ need judgments, cognitive responses, confidence in behavioral ability, and regulation intention.

### 5.1. Formation Mechanism of Family Dietary Regulation Behavioral Intention

The results show that the formation of family dietary regulation behavioral intention is not directly determined by a single factor, but is jointly affected by caregivers’ cognitive responses, family relationships, and confidence in behavioral ability. Among these factors, dietary need judgments formed by caregivers based on their own experience further influence their subsequent cognitive responses. These cognitive responses are further associated with caregivers’ judgments of dietary risk information and its potential consequences, their perceptions of caregiving responsibilities, and perceived external norms. These informational and motivational factors, in turn, show varying degrees of association with self-efficacy for dietary regulation. This is relatively consistent with the IMB framework proposed by Fisher and Fisher [36], which holds that behavior formation is usually influenced by multiple factors such as information, motivation, and behavioral ability.

Specifically, the false consensus effect significantly and positively affected cognitive dissonance (H1), indicating that caregivers’ dietary need judgments based on their own experience may further influence their cognitive evaluation of subsequent dietary arrangements. This result is somewhat consistent with Liu et al.’s [8] conclusion that the false consensus effect can cause cognitive dissonance in family dietary caregiving. In the process of family dietary caregiving, caregivers often tend to understand family members’ needs from the perspective of doing what is good for the family. When actual feedback is inconsistent with original expectations, caregivers are also more likely to re-examine current dietary arrangements and further experience cognitive dissonance. Previous research on the false consensus effect has also shown that when individuals receive external feedback that is inconsistent with their initial judgments, they may adjust their beliefs by reconsidering their perceptions of others’ views rather than relying solely on factual information when expressing opinions [37]. At the same time, cognitive dissonance further had significant positive effects on risk information perception, caregiving responsibilities, and subjective norms (H2, H3, and H4). Related research has also suggested that when individuals encounter social information that conflicts with their own judgments, such inconsistency may involve both epistemic concerns about whether their judgments are correct and relational concerns about maintaining agreement with others, belonging, and social acceptance [38]. Liu et al. further confirmed that cognitive dissonance can influence subjective norms and individual attitudes [8]. For multigenerational households, family dietary caregiving usually involves not only dietary health issues, but also family responsibility, intergenerational relationships, and emotional expression. Therefore, cognitive dissonance further promotes caregivers’ re-examination of risk information, their own responsibilities, and external expectations.

Regarding the improvement of caregiver self-efficacy, the results show that caregiving responsibilities and subjective norms both significantly and positively affected self-efficacy (H6 and H7), whereas the effect of risk information perception on self-efficacy was not supported (H5). The findings regarding caregiving responsibilities and subjective norms are consistent with those reported in previous health behavior research. Fang et al. confirmed that both intrinsic motivation and external subjective norms influence proactive health behavioral intention, with self-efficacy serving as a mediator between these motivational factors and behavioral intention [17]. Similarly, Teng et al. found that perceived social support was significantly associated with general self-efficacy among caregivers. They further suggested that social support can strengthen caregivers’ sense of responsibility, thereby encouraging more active engagement in caregiving activities [15]. Together, these findings suggest that judgments of self-efficacy are closely related to individuals’ social roles and relational contexts. In addition, previous studies have proposed that individuals can be classified into four groups according to their levels of risk perception and self-efficacy [39]. These findings provide additional insight into the differentiated relationships observed in the present study among risk information perception, motivational factors, and self-efficacy.

In addition, SE further significantly and positively affected dietary regulation behavioral intention, indicating that behavioral confidence plays a more direct role in the process of family dietary regulation. Existing studies have also pointed out that self-efficacy is one of the important factors promoting the formation of health behavior [19]. For multigenerational households, dietary arrangements usually involve differences in dietary preferences, health needs, and communication styles among members of different ages. Therefore, the more caregivers believe that they can effectively conduct family dietary communication and adjustment, the more likely they are to actively participate in family dietary regulation in the future. Previous research on family caregiving has further suggested that the caregiving experience is multidimensional [40]. The formation of family dietary regulation intention involves multiple aspects, including cognitive responses, caregiving motivation, and judgments of behavioral capability, rather than being determined by a single psychological factor [41]. Kong et al. reported that positive and negative caregiving experiences can coexist among family caregivers of older adults, with positive experiences including a sense of accomplishment, personal growth, and role satisfaction [42]. They also found that higher self-efficacy may strengthen caregivers’ confidence in coping with caregiving challenges and facilitate positive adaptation to the caregiving role [42]. Taken together, these findings suggest that understanding family dietary regulation intention requires consideration of the diverse psychological experiences of caregivers, as well as the ways in which different cognitive and motivational factors may contribute to their self-efficacy.

From the overall path structure, self-efficacy appears to play a potential mediating role between motivational factors and the intention to regulate healthy family diets. The results suggest that the relationships between caregivers’ internal sense of caregiving responsibility and perceived external normative expectations and their dietary regulation intention may be partially reflected through self-efficacy for dietary regulation. This finding is consistent with the logic of the IMB model, which proposes that motivational factors are linked to behavioral intention or behavioral outcomes through behavioral skills, and it also echoes previous studies demonstrating the mediating role of self-efficacy in the relationships among personal motivation, subjective norms, and health behavioral intention [10,17]. The theoretical significance of self-efficacy lies not only in its direct association with dietary regulation intention but also in its potential role as an important psychological pathway linking intrinsic motivation, extrinsic motivation, and dietary regulation intention.

### 5.2. IPMA Findings

The IPMA results show that the importance and performance levels of self-efficacy are both relatively high, whereas the importance and performance levels of risk information perception are both relatively low. This indicates that, compared with risk information perception alone, caregivers’ judgment of their own dietary communication and adjustment ability is more important in the formation of family dietary regulation behavioral intention.

Among the constructs, self-efficacy has the highest importance, indicating that it occupies an important position in the formation of family dietary regulation behavioral intention. The more caregivers believe that they can effectively conduct family dietary communication, coordination, and adjustment, the stronger their dietary regulation behavioral intention is. At the same time, the importance of caregiving responsibilities is also relatively high, indicating that family role responsibility also plays an important role in the process of family dietary regulation. For multigenerational households, dietary caregiving involves not only health issues, but also family responsibility and long-term relational interaction. Therefore, the relatively high-performance level of caregiving responsibilities also reflects that most caregivers in the sample already have a certain awareness of family dietary caregiving.

By contrast, the relatively low importance of risk information perception also echoes the result that it was not significantly associated with self-efficacy. This suggests that caregivers’ cognitive judgments of family dietary risk information and its potential consequences may play a relatively limited role in shaping the intention to regulate healthy family diets through the self-efficacy pathway. In addition, the performance levels of the false consensus effect, caregiving responsibilities, and self-efficacy are all relatively high, whereas the performance level of risk information perception is relatively low. This result indicates that there may be certain differences among factors in terms of importance and performance levels, and that intervention priorities need to be judged by considering both dimensions together.

Understanding these intervention priorities also requires consideration of how family dietary regulation is implemented in everyday caregiving. Family dietary regulation involves not only judgments about health risks but also the ongoing processes of meal provision, dietary planning, family communication, and the management of specific dietary issues. Lockwood et al. found that most caregivers of patients with chronic kidney disease were continuously involved in meal provision, and that their dietary support experience encompassed food literacy, social support, social responsibility, and the management of complex dietary needs [43]. Similarly, Eglseer et al. reported that providing healthy dietary support for community-dwelling older adults involves practical aspects of daily life, including communication between caregivers and care recipients, dietary appropriateness, and meal planning [44]. Together, these studies illustrate the practical and multifaceted nature of family dietary caregiving in everyday life.

Moreover, these caregiving activities are carried out within a shared family living context in which family members continuously participate in daily dietary practices [41]. Family dietary caregiving therefore involves not only the execution of specific dietary management tasks but also the ways in which family members jointly participate in and coordinate these activities. In multigenerational households, caregivers’ sense of responsibility for dietary management and their confidence in regulating family diets do not exist independently of family relationships; rather, they are developed and enacted through long-term co-residence and ongoing dietary interactions. Accordingly, the intervention priorities identified by the IPMA should be interpreted within this context of shared family participation, rather than being viewed solely as issues of individual health cognition or behavioral capability.

## 6. Conclusions

### 6.1. Research Conclusions

This study examined caregivers’ cognition of dietary imbalance behavior in multigenerational households and the psychological mechanism by which this cognition is transformed into behavioral intention to regulate healthy family diets. The results show that, in the context of dietary management in multigenerational households, internal motivation represented by caregiving responsibilities and external motivation represented by subjective norms are important factors driving caregivers’ self-efficacy in dietary regulation. Caregivers’ efficacy assessment of whether they can effectively adjust the family dietary structure is mainly positively influenced by their internally spontaneous sense of caregiving responsibility and the subjective norms in the external family context. However, risk information perception related to family members’ health did not have a significant effect on self-efficacy. This finding means that in family fields characterized by strong emotional dependence and interpersonal interaction, simply making caregivers aware of the health threats that may result from dietary imbalance does not necessarily directly provide them with practical confidence to promote family dietary adjustment and conduct intergenerational dietary communication.

Finally, the IPMA results indicate that enhancing caregivers’ self-efficacy for dietary regulation may represent an important entry point for strengthening the intention to regulate healthy family diets. Caregivers’ confidence in their ability to manage diets across generations significantly affects their behavioral intention to implement dietary structure adjustment in the future. At the same time, self-efficacy and caregiving responsibilities both show the characteristics of high importance and high performance in the overall behavioral network. Overall, the findings indicate that to facilitate the healthy transformation of family diets, public health interventions may place greater emphasis on improving caregivers’ self-efficacy in family dietary management.

### 6.2. Theoretical Contributions

First, this study shifts the research perspective by viewing dietary regulation behavior in multigenerational households as an interactive behavior embedded in family caregiving relationships rather than as a purely individual health decision. By turning attention to the interior of multigenerational households, this study finds that dietary arrangement in real life is not only an individual behavior concerned with nutritional composition, but also an important carrier through which caregivers fulfill intergenerational caregiving obligations and express emotional care. This shift in perspective helps more clearly reveal the formation mechanism of persistent unhealthy dietary habits within the family field and promotes healthier dietary arrangements.

Second, this study extends the application context of the IMB model by applying the theory to dietary regulation scenarios in complex family interpersonal fields. This study applies the IMB model to the context of dietary regulation in multigenerational households and examines the roles of information, motivation, and behavioral skills in the formation of behavioral intention from the perspective of family caregiving relationships. It provides a new reference for applying the IMB model in family health behavior research.

Third, this study supplements research on the formation mechanism of family dietary caregiving behavioral intention. Liu et al. studied why family food provision behavior continues even when it is known to be potentially unreasonable [8]. From a different perspective, the present study explores how caregivers form dietary arrangements based on dietary judgments that they consider reasonable under the premise of doing what is good for the family, and how they gradually form future regulation intentions through subsequent feedback. It ultimately extends discussion to the formation process of psychological reflection and willingness to correct diets triggered by subjective misjudgment of needs.

Finally, this study further clarifies the potential mediating role of self-efficacy between motivational factors and the intention to regulate healthy family diets. By integrating family role responsibilities, normative expectations, and judgments of behavioral capability within a unified theoretical framework, the study further highlights the theoretical significance of self-efficacy in linking family caregiving motivations with the formation of dietary regulation intention. In this regard, the findings provide a more specific account of the role of the behavioral skills component of the IMB model in the context of complex family relationships and add theoretical evidence for understanding the formation of healthy dietary regulation intention in multigenerational households.

### 6.3. Managerial Importance

The structural model and IPMA results jointly show that, compared with risk information perception, caregiving responsibilities and subjective norms have higher importance in the formation of subsequent behavioral intention. This indicates that in the practice of family dietary regulation, merely increasing caregivers’ attention to dietary risks may not be sufficient to promote subsequent regulation behavior. For relevant management departments, greater attention may be paid to the role of family responsibility awareness and normative expectations in family dietary management.

The structural model results show that cognitive dissonance is significantly associated with risk information perception, caregiving responsibilities, and subjective norms. This indicates that psychological responses related to family dietary arrangements are not limited to a single factor, but simultaneously involve multiple aspects such as risk information perception, responsibility, and normative expectations. Therefore, when conducting family health promotion work, interventions may avoid focusing on only one single factor and should instead attend to the synergistic effects among different factors.

The IPMA results show that self-efficacy has the highest importance, indicating that caregivers’ confidence in their own ability to regulate family diets is an important factor promoting the formation of subsequent behavioral intention. Therefore, in family health promotion practice, further attention may be paid to building caregivers’ confidence in their dietary regulation ability, so as to increase the likelihood of implementing family dietary regulation measures.

### 6.4. Limitations and Future Research

Although this study reveals the formation mechanism of dietary regulation behavioral intention in multigenerational households, there remains room for further extension.

First, this study mainly analyzes the relationships among caregiver need judgment, cognitive response, and behavioral intention based on cross-sectional data, whereas family dietary regulation itself is often continuous and dynamic. Future research may further incorporate longitudinal research designs to observe the adjustment process of family dietary arrangements in long-term interaction and how feedback from family members continuously affects caregivers’ subsequent judgments and behavioral changes.

Second, this study mainly analyzes the issue from the perspective of caregivers, whereas dietary arrangements in multigenerational households usually involve negotiation and interaction among multiple actors, including older adults, parents, and children. Future research may further combine bidirectional data from family members, family interviews, or real shared-meal contexts to conduct deeper discussion of the dietary negotiation process in intergenerational interaction, thereby further enriching contextual understanding in family dietary behavior research.

In addition, this study mainly focuses on family dietary regulation behavioral intention, whereas actual dietary behavior may also be influenced by factors such as family structure, income level, and long-term living habits. Future research may further conduct comparative studies across different family types and life contexts, and further attend to the process through which behavioral intention is transformed into long-term actual behavior, so as to deepen understanding of the formation mechanism of family dietary regulation behavioral intention.

Finally, although the online survey was distributed across different regions of China and efforts were made to include participants with diverse sociodemographic characteristics, the sample may still have been influenced by the recruitment approach and participants’ self-selection. Therefore, the generalizability of the findings to other regions, family structures, and sociocultural contexts should be interpreted with caution. Future research could further expand the geographic coverage and diversity of the sample and validate the proposed model across a broader range of multigenerational households to examine the robustness and generalizability of the findings.

## Figures and Tables

**Table 1:** Measurement Items.

Construct	Operational Definition	Items	Source
False consensus effect/FCE	The caregiver’s tendency to infer family members’ dietary needs based on his or her own experience and subjective understanding.	FCE1: I judge what and how much my family members should eat recently based on my own past dietary experience.	[8]
		FCE2: I care deeply about my family members and cannot help paying attention to them, fearing that they may have dietary problems.	
		FCE3: Caring about whether my family members have eaten enough and eaten well makes me feel that I have a better grasp of their current dietary situation.	
Cognitive Dissonance/CD	The psychological tension caregivers experience between the caregiving belief of doing what is good for the family and actual feedback that does not meet expectations.	CD1: I originally do this for my family members’ good, but when the effect is not as expected, I sometimes feel somewhat uneasy about the current dietary arrangements.	[8]
		CD2: I originally do this for my family members’ good, but when the effect is not as expected, I become somewhat uncertain whether the current dietary arrangements are appropriate.	
		CD3: I originally do this for my family members’ good, but when the effect is not as expected, I usually think about whether the current dietary arrangements fully meet their needs.	
		CD4: I originally do this for my family members’ good, but when the effect is not as expected, I usually think about whether the current dietary arrangements need to be adjusted.	
Risk Information Perception/RIP	The cognitive judgments and risk concerns caregivers form about possible risks in the family’s current dietary status and its potential health consequences after receiving feedback information related to family diets.	RIP1: I worry that the current dietary status of my family members may be unhealthy.	[21,22]
		RIP2: I worry that continuing to maintain this dietary status that is not as expected may harm my family members’ health.	
		RIP3: I worry that older adults and children are more likely to be adversely affected under this dietary status.	
		RIP4: If no intervention is made in the family’s current dietary status, there may be potential risks.	
Caregiving Responsibilities/CR	The internal moral driving force formed by caregivers based on family roles, filial beliefs, and caregiving responsibilities.	CR1: I think I should take care of my other family members to some extent.	[23,24]
		CR2: I feel that my family members need my care and attention in diet and daily life.	
		CR3: I often naturally care about my family members’ dietary status.	
		CR4: I often actively communicate with my family members about their dietary status to understand their thoughts and feelings.	
Subjective Norms/SN	The normative expectations caregivers perceive in the family, derived from family roles, intergenerational relationships, and surrounding social beliefs.	SN1: Most of my family members hope that I will help take care of them.	[8]
		SN2: As a child or parent, I sometimes feel that social beliefs create certain expectations for me to take good care of my family members’ diet and daily life.	
		SN3: Most important people around me believe that, as a child or parent, one should take good care of family members’ diet and daily life.	
Self-Efficacy/SE	Caregivers’ behavioral confidence in whether they can effectively conduct family dietary communication, coordination, decision-making, and problem solving when regulating healthy family diets.	SE1: I am confident that I can promote healthier dietary arrangements in my family.	[25]
		SE2: If I make enough effort, I can usually find ways to promote effective dietary communication among family members.	
		SE3: Even when time or energy is limited, I can find ways to adjust the family dietary situation.	
		SE4: When I encounter some problems within the family, I can usually make my own judgment.	
Dietary Regulation Behavioral Intention/DRBI	Caregivers’ behavioral willingness in future family dietary contexts to actively communicate about and adjust inappropriate dietary arrangements in the family and to continuously guide family members toward more appropriate dietary patterns.	DRBI1: I am willing to actively communicate with my family members about their healthy dietary needs.	[16,26]
		DRBI2: I plan to adjust inappropriate dietary arrangements according to the actual situation.	
		DRBI3: In the near future, I will consider continuously guiding my family members to form more appropriate dietary patterns.	

**Table 2:** Demographic Information (n = 467).

Variable	Group	Frequency	Ratio (%)
Gender	Male	193	41.33%
	Female	274	58.67%
Age	20–30 years old	132	28.27%
	31–45 years old	292	62.53%
	46 years old and above	43	9.21%
Education	Junior high school or below	10	2.14%
	High school/technical secondary school	45	9.64%
	Bachelor’s degree/junior college	382	81.80%
	Graduate degree or above	30	6.42%
Annual household income level	Above the local average	96	20.56%
	Comparable to the local average	352	75.37%
	Below the local average	19	4.07%
Total		467	100%

**Table 3:** Measurement Model Analysis Results.

Construct	Items	Standardized Outer Loadings	Cronbach’s α	Composite Reliability/CR	Average Variance Extracted/AVE
FCE	FCE1	0.715	0.700	0.833	0.626
	FCE2	0.831			
	FCE3	0.823			
CD	CD1	0.789	0.796	0.867	0.620
	CD2	0.795			
	CD3	0.794			
	CD4	0.772			
RIP	RIP1	0.825	0.875	0.915	0.728
	RIP2	0.878			
	RIP3	0.857			
	RIP4	0.853			
CR	CR1	0.768	0.752	0.843	0.574
	CR2	0.751			
	CR3	0.709			
	CR4	0.800			
SN	SN1	0.818	0.717	0.841	0.638
	SN2	0.728			
	SN3	0.847			
SE	SE1	0.822	0.773	0.855	0.596
	SE2	0.755			
	SE3	0.773			
	SE4	0.735			
DRBI	DRBI1	0.791	0.745	0.855	0.662
	DRBI2	0.813			
	DRBI3	0.836			

* The meaning of the variables: False consensus effect (FCE); Cognitive Dissonance (CD); Risk Information Perception (RIP); Caregiving Responsibilities (CR); Subjective Norms (SN); Self-Efficacy (SE); Dietary Regulation Behavioral Intention (DRBI).

**Table 4:** Discriminant validity results: HTMT.

	DRBI	CD	CR	FCE	RIP	SE
CD	0.656					
CR	0.896	0.654				
FCE	0.786	0.676	0.828			
RIP	0.381	0.507	0.439	0.31		
SE	0.894	0.577	0.868	0.795	0.276	
SN	0.755	0.603	0.895	0.797	0.384	0.823

* The meaning of the abbreviations: False consensus effect (FCE); Cognitive Dissonance (CD); Risk Information Perception (RIP); Caregiving Responsibilities (CR); Subjective Norms (SN); Self-Efficacy (SE); Dietary Regulation Behavioral Intention (DRBI); HTMT: Heterotrait–monotrait ratio of correlations.

**Table 5:** Discriminant validity results: Fornell–Larcker criterion.

	DRBI	CD	CR	FCE	RIP	SE	SN
DRBI	**0.814**						
CD	0.507	**0.787**					
CR	0.671	0.508	**0.758**				
FCE	0.57	0.506	0.605	**0.791**			
RIP	0.309	0.423	0.357	0.245	**0.853**		
SE	0.681	0.453	0.662	0.585	0.226	**0.772**	
SN	0.555	0.461	0.656	0.567	0.305	0.618	**0.799**

* Note: The diagonal (bold) elements are the square roots of AVE. The meaning of the abbreviations: False consensus effect (FCE); Cognitive Dissonance (CD); Risk Information Perception (RIP); Caregiving Responsibilities (CR); Subjective Norms (SN); Self-Efficacy (SE); Dietary Regulation Behavioral Intention (DRBI).

**Table 6:** Structural Model Analysis Results.

Constructs	R^2^	Q^2^
Cognitive Dissonance/CD	0.256	0.248
Risk Information Perception/RIP	0.179	0.056
Caregiving Responsibilities/CR	0.258	0.240
Subjective Norms/SN	0.212	0.206
Self-Efficacy/SE	0.499	0.179
Dietary Regulation Behavioral Intention/DRBI	0.464	0.125

* The meaning of the abbreviations: R-square (R^2^); Stone-Geisser Q^2^ (Q^2^).

**Table 7:** Path Analysis Test Results.

Hypothesis	Path	Std Beta	*p*-Value	VIF	Results
H1	FCE → CD	0.506	0.000	1.000	Support
H2	CD → RIP	0.423	0.000	1.000	Support
H3	CD → CR	0.508	0.000	1.000	Support
H4	CD → SN	0.461	0.000	1.000	Support
H5	RIP → SE	−0.039	0.297	1.158	No Support
H6	CR → SE	0.462	0.000	1.843	Support
H7	SN → SE	0.327	0.000	1.774	Support
H8	SE → DRBI	0.681	0.000	1.000	Support

* The meaning of the abbreviations: False consensus effect (FCE); Cognitive Dissonance (CD); Risk Information Perception (RIP); Caregiving Responsibilities (CR); Subjective Norms (SN); Self-Efficacy (SE); Dietary Regulation Behavioral Intention (DRBI). Standardized Path Coefficient (Std Beta); Variance Inflation Factor (VIF).

## Data Availability

The original contributions presented in this study are included in the article. Further inquiries can be directed to the corresponding author.
